# A National survey of surgeons’ perspectives on the treatment of adults with acute appendicitis

**DOI:** 10.1007/s00423-025-03960-w

**Published:** 2026-01-08

**Authors:** Morten T. Grundahl, Eskil S. Bohr, Thomas K. Jensen, Morten L. Lauritsen, Liv BJ Nielsen, Anders Peter Skovsen, Henry G. Smith

**Affiliations:** 1 Abdominalcenter K, Copenhagen University Hospital – Bispebjerg and Frederiksberg, Copenhagen, Denmark; 2 Department of Surgery, Zealand’s University Hospital, Køge, Denmark; 3 Department of Gastroenterology, Copenhagen University Hospital – Herlev, Herlev, Denmark; 4 Department of Surgery, Copenhagen University Hospital – Hvidovre, Hvidovre, Denmark; 5 Department of Surgery, Copenhagen University Hospital – Nordsjaelland, Hillerød, Denmark; 6 Department of Clinical Medicine, Copenhagen University, Copenhagen, Denmark

**Keywords:** Acute appendicitis, Acute care surgery, General surgery, Treatment preferences

## Abstract

**Aims:**

Management of patients with suspected acute appendicitis (AA) varies and often depends on the admitting surgeon’s preferences. Here we investigated surgeons’ perspectives on the management of AA and their own preferences if they themselves were admitted with suspected AA.

**Methods:**

A 17-point web-based questionnaire was distributed to all acute general surgical departments in Denmark over a 1-month period. Four items regarded demographics, 9 items addressed the treatment of patients with suspected AA and the last 4 items explored surgeons’ personal treatment preferences.

**Results:**

213 complete responses were received, with most respondents < 50 years old (86%). Most respondents rarely or never use scoring systems (77%) when assessing patients with suspected AA. Most felt pre-operative imaging wasn’t indicated in patients < 50 years with typical presentations (4%), although this rose in the case of atypical presentations (56%). Less than 5% routinely discuss non-operative management with patients who are otherwise fit for surgery. In afebrile patients with AA, 9% would operate during the night based on a clinical diagnosis, rising to 21% if the diagnosis was confirmed on imaging, and to 49% if the patient also had raised inflammatory markers. Regarding preferences for their own treatment, only a minority would want to be assessed using scoring systems (13%), whereas almost half would want pre-operative imaging (47%).

**Conclusion:**

Considerable variation is noted in surgeons’ perspectives on the management of patients with AA. Discrepancies are noted in surgeons’ routine clinical practice and their preferences for their own treatment, particularly regarding pre-operative imaging.

**Supplementary Information:**

The online version contains supplementary material available at 10.1007/s00423-025-03960-w.

## Introduction

Acute appendicitis is the most common acute general surgical condition worldwide, with an estimated lifetime risk of up to 8% [1, 2]. Despite its frequency, the management of patients with suspected appendicitis is anything but standardized. Although both national and international consensus guidelines are available, adherence to these recommendations is questionable [3–6]. Several studies have demonstrated variations in management at both national and international levels [7–9]. As such, one could argue that surgical dogma still dominates the decision-making process in this patient group in many institutions.

This inconsistency is perhaps unsurprising given the plethora of controversies that exists regarding the diagnosis and treatment of patients with suspected appendicitis. For many, appendicitis remains a clinical diagnosis, where laparoscopy can be used both as a diagnostic and therapeutic tool. Others advocate for pre-operative imaging, either in the form of ultrasound (US) or computed tomography (CT), to confirm the diagnosis first, with laparoscopy reserved as a therapeutic intervention. Routine use of CT presents another issue as it may lead to overtreatment of patients with uncomplicated spontaneous resolving appendicitis [10]. Magnetic resonance imaging (MRI) is an alternative to CT, with high sensitivity and specificity, whilst avoiding radiation [11]. However, its widespread use is limited by higher costs and poorer availability compared to other imaging modalities. Risk prediction models may also be used to improve diagnosis and stratify patients to either imaging or operation [12, 13]. However, a multitude of scoring systems are available, some of which are inaccurate and poorly validated [7, 14]. Once a diagnosis is made, most surgeons still regard appendicitis as a surgical emergency, necessitating urgent intervention. However, in selected patients, there is evidence to suggest that appendicectomy can be safely postponed until the next day or even avoided altogether and treated with antibiotics instead [15, 16].

Due to these different possible approaches in managing patients with suspected appendicitis opinions vary a great deal. However, the extent of this variation between surgeons is relatively unknown. Furthermore, little is known regarding surgeons’ preferences for their own treatment if they were admitted with suspected appendicitis and how that compares to their normal clinical practice. Here we performed a national questionnaire-based study to investigate surgeons’ perspectives regarding the management of patients with suspected appendicitis and their own preferences if they themselves were admitted with symptoms compatible with acute appendicitis.

## Methods

This was a survey-based study and is reported according to CROSS (Checklist for Reporting of Survey Studies) guidelines [17]. A 17-point questionnaire was developed by the study group comprising abdominal surgeons from university and regional hospitals in Denmark, all actively involved in the management of acute appendicitis. The questionnaire consisted of three parts (Supplementary appendix 1). The first part included 4 questions regarding the respondents’ age, career stage and place of work. The second part included 9 questions regarding the respondents’ normal clinical practice in treating patients with suspected appendicitis. The third part consisted of 4 questions regarding the respondents’ preferences for their own treatment if they were admitted with suspected appendicitis.

The target population for this questionnaire was surgeons of any career stage currently working in hospitals that manage adult patients with suspected appendicitis. A pilot test of the questionnaire was performed in a group of 10 surgeons of varying career stages to ensure clarity and relevance before distribution. The survey was web-based and hosted using REDCap (Research Electronic Data Capture; Vanderbilt University, Nashville, TN, USA) through the hospital regions’ secure server. A link to the survey was distributed to the head of department of every acute general surgical hospital in Denmark, as well as via the Danish Society of Young Surgeons newsletter and on social media. A single study period was used, where the survey was open for 1 month between 31/01/25–28/02/25. No follow-up messages or reminders were used. The survey link included a short statement on the goals of the survey and clarified that all responses would be anonymous. IP addresses or other personal identifiers were not collected. The full survey was only accessible to respondents who reported that they work at a hospital providing care for adults with suspected appendicitis. Other respondents were excluded after this initial question. Approvals regarding data privacy were obtained from the Danish Data Protection Agency (P-2024–17937). Formal ethical approval was not required for this study due to national regulations exempting questionnaire-based research among healthcare professionals from ethics committee review.

After the survey was closed, all responses were collated. Survey responses that were not entirely complete were included in the analyses where fields were completed but excluded from those that were not completed. Descriptive statistics were first performed using the entire dataset. Comparative statistics using the Chi-square test were then performed according to respondent age, career stage and the region in which they work. Career stages were defined as follows: HO – house officer; CT – core trainee; SpR – specialist registrar; JC – junior consultant; SC – senior consultant. P values < 0.05 were considered statistically significant. All analyses were performed using Graphpad Prism, version 10.0.

## Results

The survey page was accessed a total of 251 times during the study period, with a total of 213 responses. A summary of the respondents’ characteristics is provided in Table 1. Responses were received from 21 out of 26 different hospitals, from 4 of the 5 Danish regions. Most respondents were from The Capital Region (54.9%), and the majority were under 50 years of age (86.4%). The survey was completed by 35 (16.4%) house officers, 50 (23.5%) core trainees, 49 (23.0%) specialist registrars, 35 (16.4%) junior consultants, 40 (18.8%) senior consultants and 4 (1.9%) who did not specify their career stage. Thus, responses represented all levels of surgical training across Denmark.

**Table 1:** Characteristics of the survey respondents

		Number (%)
**Total respondents**		213
**Region**	**Capital**	117 (54.9)
	**Zealand**	26 (12.2)
	**South**	40 (18.8)
	**Mid-Jutland**	28 (13.1)
	**Not specified**	2 (0.9)
**Grade**	**House officer**	35 (16.4)
	**Core trainee**	50 (23.5)
	**Specialist registrar**	49 (23.0)
	**Junior consultant**	35 (16.4)
	**Senior consultant**	40 (18.8)
	**Not specified**	4 (1.9)
**Age (years)**	**< 30**	45 (21.1)
	**30–40**	103 (48.4)
	**40–50**	36 (16.9)
	**> 50**	25 (11.7)
	**Not specified**	4 (1.9)

The regions correspond to the 5 Danish healthcare regions. No responses were received from the North Jutland region

## Access to diagnostic imaging

**Fig. 1:**
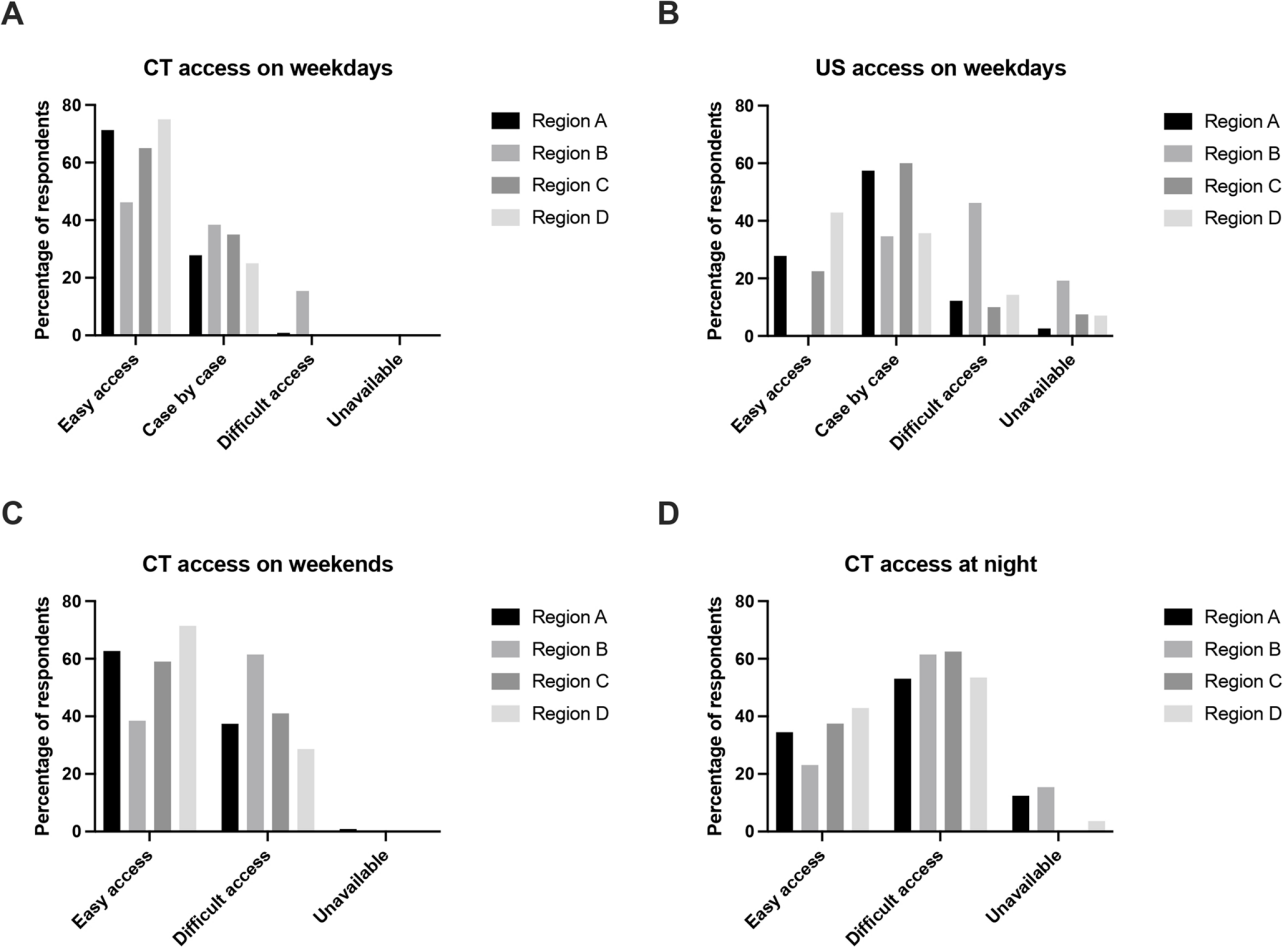
Regional variation in access to imaging modalities in the assessment of patients with suspected appendicitis. (**A**) Computed tomography (CT) access during working hours on weekdays (p < 0.001, Chi-square test). (**B**) Ultrasound (US) access during working hours on weekdays (p < 0.001, Chi-square test). (**C**) CT access during daytime hours on weekends (p = 0.265, Chi-square test). (**D**) CT access during the night (p = 0.159, Chi-square test)

Across all responses, CT was generally more accessible than radiology-performed abdominal ultrasound (US) during daytime hours for the assessment of patients with suspected appendicitis (reported as easily accessible by 67.5% for CT versus 25.2% for US). Statistically significant regional variations were observed in the availability of both modalities (Fig. 1a, b). Only a minority of respondents (36.2%) reported that abdominal US was routinely available for these patients during the weekends. While CT availability for the assessment of patients with suspected appendicitis remained largely unchanged during weekends, it was less accessible out-of-hours on weekdays. However, no statistically significant regional variation was observed in this context (Fig. 1c, d).

## Use of appendicitis scoring systems

The majority of respondents stated that they rarely (27.2%) or never (49.8%) use appendicitis scoring systems when assessing patients with suspected appendicitis. Just over 5% of respondents stated that they regularly (4.3%) or always (1.0%) used these scoring systems. No statistically significant differences in the use of scoring systems were noted according to career stage or the region of respondents.

## Indications for pre-operative imaging

**Fig. 2:**
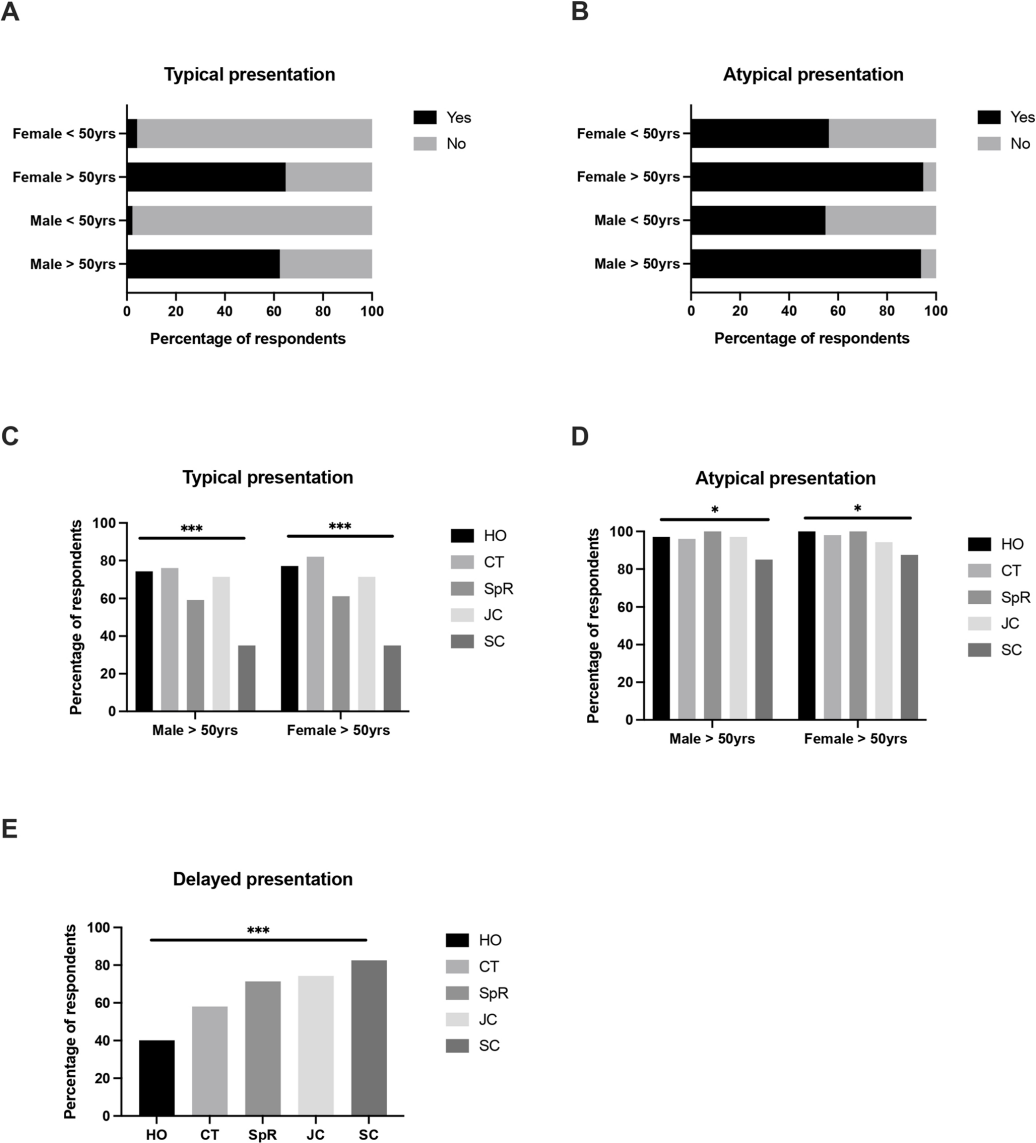
Indications for pre-operative imaging in patients with (**A**) typical or (**B**) atypical presentations for acute appendicitis. Variations in indications for imaging according to surgeon’s career stage in patients with (**C**) typical, (**D**) atypical, or (**E**) delayed presentations for acute appendicitis. HO– house officer; CT – core trainee; SpR – specialist registrar; JC – junior consultant; SC – senior consultant. * = p < 0.05;** = p < 0.01, *** = p < 0.001

Opinions regarding the need for pre-operative imaging were then investigated using a range of different clinical scenarios based on patient age and sex (Fig. 2). In patients with typical presentations of acute appendicitis, the majority of respondents stated that imaging was needed in patients aged > 50 years (62.4% for male patients, 64.8% for female patients), whereas very few felt this was needed in those aged < 50 years (2.3% for male patients, 4.2% for female patients). In the case of atypical presentations, almost all respondents stated that pre-operative imaging was needed in patients aged > 50 years (93.9% for male patients, 94.8% for female patients), with the majority also stated imaging was needed in those < 50 years (54.9% for male patients, 56.3% for female patients). The majority of respondents also stated that imaging was indicated in patients with delayed presentations (> 3 days, 64.3%), whereas only 17.8% stated that markedly elevated inflammatory markers (CRP > 100) was an indication for pre-operative imaging.

Statistically significant differences in the respondents’ reported indications for pre-operative imaging were noted according to career stage (Fig. 2). Here, the greatest differences were observed in older patients with typical presentations, where those with earlier career stage were more likely to state that imaging was indicated (75.7% of house officers versus 35.0% of senior consultants), and in patients with delayed presentations, where those with later career stages were more likely to state that imaging was indicated (82.5% of senior consultants versus 40.0% of house officers).

## Indications for operation during the night

**Fig. 3:**
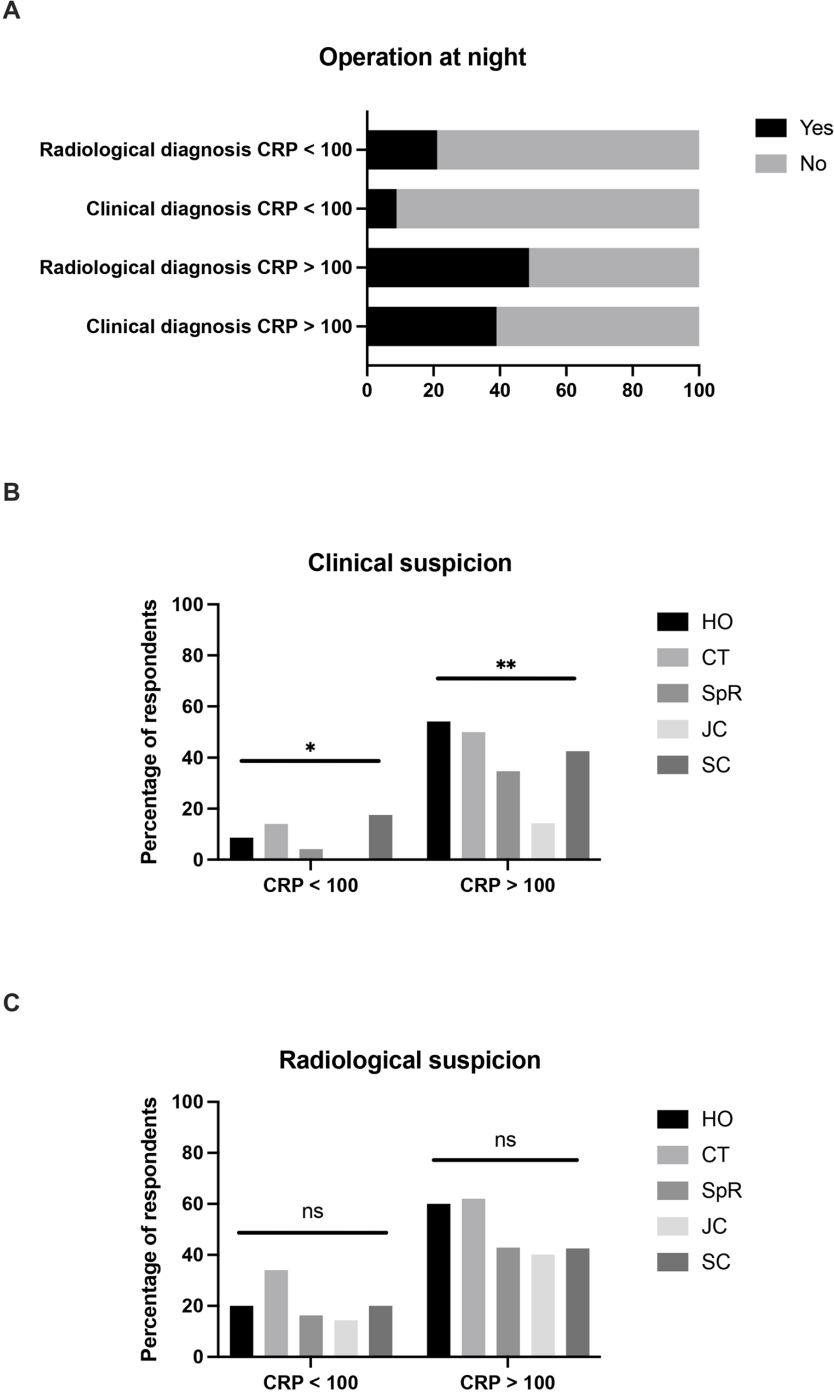
(**A**) Indication for operations after midnight in patients with suspected acute uncomplicated appendicitis. The percentage of respondents opting for operation are as follows: radiological diagnosis, CRP < 100 – 21.1%; clinical diagnosis, CRP < 100 – 8.9%; radiological diagnosis, CRP > 100 – 48.8%; clinical diagnosis, CRP > 100 – 39.0%. Variations in indication for operations after midnight according to surgeons’ career stage in patients with (**B**) clinical or (**C**) radiological suspicion of appendicitis. CRP – c reactive protein; HO – house officer; CT – core trainee; SpR – specialist registrar; JC – junior consultant; SC – senior consultant. * = p < 0.05; ** = p < 0.01

Respondents were then asked to state in which scenarios they felt operations at night would be indicated for patients with suspected acute uncomplicated appendicitis, in the absence of fever or signs of generalized peritonitis. In afebrile patients with low inflammatory markers (CRP < 100), 8.9% of respondents would operate overnight based on clinical suspicion of acute appendicitis, rising to 21.1% if the diagnosis had been confirmed with pre-operative imaging. In contrast, in patients with markedly elevated inflammatory markers (CRP > 100), 39.0% of respondents would operate during the night based on clinical suspicion of acute appendicitis, rising to 48.8% if the diagnosis had been confirmed with pre-operative imaging. Again, statistically significant differences in the indications for surgery were noted according to career stage, where those with an earlier career stage were more likely to state that they would operate on patients during the night (Fig. 3).

### Removal of a macroscopically normal appendix

Respondents were then asked if they would remove a macroscopically normal appendix during a laparoscopic operation for presumed appendicitis. Here, 11.2% of respondents stated they would remove the appendix if no other cause for abdominal pain was found, whereas 50.0% would only remove it if an agreement to do so had been made with the patient before surgery. Of the remaining respondents, 3.4% stated they would remove the appendix regardless of the intra-operative findings, whereas 35.4% of respondents stated they would leave the appendix in situ. No differences in responses were noted according to career stage or region.

## Non-operative treatment

The majority of respondents stated that they rarely (27.2%) or never (49.8%) discuss the option of non-operative management with patients diagnosed with acute appendicitis who are judged to be candidates for surgery. Less than 5% of respondents stated that they regularly (3.4%) or always (0.5%) discuss this option with these patients. No differences in responses were noted according to career stage or region.

## Surgeons’ perspectives on their own treatment

**Fig. 4:**
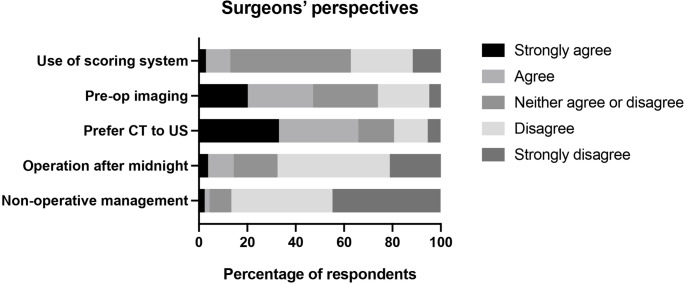
Surgeons’ perspectives on their own treatment if they were admitted with suspected appendicitis. CT – computed tomography; US – ultrasound.

Finally, respondents were asked to state how they would wish to be assessed and treated if they were themselves admitted with suspected appendicitis (Fig. 4). Only a minority of respondents stated that they would want to be assessed using an appendicitis scoring system (13.0% indicated agreement: strongly agree 2.9%, agree 10.1%), whereas almost half stated that they would want pre-operative imaging (47.1% indicated agreement: strongly agree 20.2%, agree 26.9%). If pre-operative imaging were to be performed, the majority of respondents would prefer CT to an abdominal ultrasound (65.9% indicated agreement: strongly agree 33.2%, agree 32.7%). The pattern of responses was broadly unchanged when only respondents aged < 50 years were considered, with 47.3% stating that they would want pre-operative imaging and 65.2% preferring CT to abdominal ultrasound.

The majority of respondents would not want to be operated on after midnight (67.5% indicated disagreement: strongly disagree 46.4%, disagree 21.1%) if they were admitted with acute uncomplicated appendicitis. Furthermore, an overwhelming majority stated that they would not want to undergo non-operative treatment (86.5% indicated disagreement with conservative management: strongly disagree 44.7%, disagree 41.8%).

## Discussion

This national questionnaire study highlights substantial variation in how surgeons across Denmark assess and manage patients with suspected appendicitis. Variation was seen in the availability and use of diagnostic imaging, use of scoring systems, timing of surgical intervention, and attitudes toward non-operative treatment. Such variations are not a local phenomenon, with both national and international studies demonstrating similar differences in practice within these controversial topics [18–20]. However, the current study also demonstrated a marked discrepancy between surgeons’ stated clinical practice and their preferences for personal treatment if they were to present with suspected appendicitis themselves.

The role of imaging continues to be a focal point of debate in the diagnostic workup of suspected appendicitis [6, 21]. Pre-operative imaging is generally accepted in older patients due to the increased incidence of other pathologies, such as diverticular disease and colon cancer [22, 23]. However, different cutoffs for defining an older population have been proposed. Perhaps due to the increased incidence of early onset colon cancer, newer guidelines differentiate diagnostic pathways for patients above or below a cutoff of 40 years [5, 6, 24]. It is interesting to note that despite the use of a more conservative cutoff of 50 years, the current study found that over a third of surgeons do not believe pre-operative imaging is indicated in older patients with typical presentations. Pre-operative imaging also has value in identifying patients with periappendicular abscesses, in whom percutaneous drainage and systemic antibiotics is an option instead of surgery. Periappendicular abscesses should be suspected in patients with delayed presentations or markedly raised inflammatory markers [5]. However, in the current study, more than a third of respondents would not image patients with delayed presentations, and less than 20% would image patients with a CRP > 100. These findings indicate persistent variation in clinical thresholds for imaging, even among experienced surgeons, perhaps indicative of the ongoing influence of local training and culture.

A further controversy with regards to pre-operative imaging is whether it should be performed in all patients, as recommended in the national guidelines of other countries, or if patients should be stratified to imaging based on appendicitis scoring systems, as recommended in others [3, 5]. Routine use of pre-operative imaging can lead to several issues such as increased costs, strain on radiological resources, unnecessary radiation exposure, and potential overtreatment of this patient group. The DIAMOND trial randomised patients with early equivocal appendicitis to either imaging or observation and found that the rates of intervention were higher in those allocated to the imaging arm when compared to the observation arm (72% versus 57%, *p* = 0.032) [10]. This suggests that in some patients, appendicitis resolve spontaneously without the need for intervention. The use of scoring systems to rationalise the use of imaging is an alternative strategy, although a multitude of these systems have been developed. The performance of various scoring systems was recently assessed in the RIFT study, which found the Adult Appendicitis Score (AAS) to perform best in women and the Appendicitis Inflammatory Response Score (AIRS) to perform best in men [7]. These models can be used to allocate patients to different pathways, where low-risk patients can be discharged, high-risk patients can be booked for surgery and imaging can be reserved for those with intermediate risk, as suggested by the recently published Swedish national guidelines [5]. However, the current study also found that the majority of respondents neither used these systems routinely in their own clinical practice, nor would want to be assessed using them if they presented with appendicitis themselves. The reasons for this are unclear as convincing arguments can be made for the adoption of a validated and user-friendly scoring system as a relatively low-cost, scalable intervention that could improve diagnostic consistency, rationalise the use of pre-operative imaging and reduce inter-clinician variability. The relatively easy access to operating theatres in Denmark may partly explain why respondents reported less emphasis on patient stratification or high negative predictive thresholds before proceeding to surgery but other cultural factors regarding the perceived value of scoring systems compared to clinical judgement may also play a role. In healthcare systems with more limited surgical resources, a stricter stratification may be necessary to avoid unnecessary operations. Interestingly the more restrictive approach to pre-operative imaging in Denmark does not seem to impact on the negative appendicectomy rate. In a single centre retrospective study where < 20% of patients aged between 16 and 45 years had pre-operative imaging, the negative appendicectomy rate was 6.6%, in keeping with internationally reported rates [25].

Beyond whether to image, the choice of imaging modality is also a subject of contention. Our data show that CT is generally more accessible than US, particularly during weekends and overnight. While CT is more accurate than US in diagnosing simple and complicated appendicitis [26], it comes with the trade-off of radiation exposure and associated risks, especially in younger patients [27, 28]. A recent study projected that approximately 5% of all newly diagnosed cancers could be attributed to radiation exposure associated with the use of CT scanning [29]. Nonetheless, many respondents showed a clear preference for CT over US, both in clinical practice and for themselves. Regional variation in the accessibility of both imaging modalities was evident, suggesting that logistical limitations rather than clinical preference may be influencing diagnostic pathways. If the diagnostic approach to this common surgical condition is to be standardized, then addressing discrepancies in access to imaging services will be an essential first step.

Significant variation was also noted in the perceived need for surgery during nighttime hours. Concerns about delaying surgery are based on the belief that the severity of appendicitis progresses with time, leading to increased risks of perforated appendicitis and further complications. However, an increasingly popular view of appendicitis is that it comprises a spectrum of different pathological processes, ranging from those cases that resolve spontaneously and those that lead to perforation within a few hours of the onset of symptoms [30, 31]. A meta-analysis of 11 non-randomised studies, which included over 8,000 patients with acute appendicitis, found that in-hospital delays of up to 24 h were not associated with increased risks of perforation [32]. These data are supported by the recent DELAY trial, which randomised 127 patients with image-confirmed appendicitis to either immediate (within 6 h) or delayed (after 6 am the next day) appendicectomy [33]. Patients allocated to the delayed group had a lower rate of 30-day post-operative complications (10.2% versus 22.4%, *p* = 0.07), which met the study’s primary endpoint for demonstrating non-inferiority of delayed appendicectomy. Current guidelines reflect these data, stating that delays of up to 24 h are acceptable in patients with uncomplicated appendicitis [5, 34]. Despite this evidence nearly half of respondents in the current study indicated they would still proceed with surgery during the night if imaging confirmed the diagnosis and CRP > 100. Interestingly, the likelihood of opting for night-time surgery was higher among surgeons earlier in their careers. This could reflect broader adoption of the most recent evidence detailed above amongst more experienced clinicians. However, other potential explanations include cultural factors such as avoiding perceived underperformance, limited daytime operative capacity, critique during morning handovers, or by educational motivations whereby junior surgeons may be more eager to take operative opportunities. Importantly, this decision also appeared to be influenced by the availability of pre-operative imaging and inflammatory markers. These findings highlight the complexity of surgical decision-making, where clinical, institutional, and personal factors converge.

Perhaps the most intriguing aspect of the study lies in the gap between what surgeons report doing for their patients and what they would want for themselves. For instance, nearly half of the respondents under 50 years of age stated they would want pre-operative imaging if admitted with suspected appendicitis, compared to less than 5% who stated they routinely image patients in the same age group. Similarly, a majority would not want to be operated on during night hours, despite a substantial number indicating they would proceed with surgery under such circumstances for their patients. This dissonance suggests that surgeons, when placed in the hypothetical role of the patient, adopt a more cautious and individualized approach, perhaps better aligned with emerging evidence and best practices. This degree to which this difference may be explained by perceived institutional pressures, time constraints, or clinical inertia is unclear. However, these findings raise interesting ethical questions regarding the gap between what a clinician judges to be acceptable for their patients and what their own preferences for treatment are. It is interesting to speculate whether patients would have similar preferences regarding their treatment if they were fully informed on the most recent data and convincing arguments can be made for increased adoption of shared decision making in this context.

In contrast to the variability seen in other areas, attitudes toward non-operative management of appendicitis were more consistent. Most respondents reported rarely or never discussing antibiotic-only treatment with eligible patients and similarly indicated they would not opt for it themselves. This reluctance is perhaps not unfounded. Early randomised trials of non-operative management with antibiotics as an alternative to appendicectomy in patients with acute uncomplicated appendicitis appeared to demonstrate benefits in terms of reducing treatment-related complications [35, 36]. However, further studies have demonstrated issues with a non-operative approach, particularly with regard to readmissions with recurrent appendicitis. With long-term follow-up from the APPAC trial, non-operative treatment was associated with a 1-year recurrence rate of 27.3%, rising to 39.1% after 5-year follow-up [37]. Similar rates were also described in the CODA trial, which also demonstrated higher risks of recurrence in patients with appendicoliths, who had a 90-day recurrence rate of over 40% [38]. A recent meta-analysis of 8 randomised trials comprising over 3,000 patients found that non-operative management had no benefit in terms of complication rates and was associated with a 6-fold increase in the risk of hospital re-admission within 1-year of diagnosis [39]. To further complicate the issue of non-operative management, 2 randomised trials have demonstrated similar rates of resolution in patients with acute uncomplicated appendicitis that were either treated with antibiotics or placebo [40, 41]. While treatment with antibiotics can be considered in situations where surgery is not possible due to patient or environmental factors, these findings highlight the current difficulties in defining the most appropriate treatment strategy, particularly for patients with uncomplicated appendicitis. Current guidelines currently still recommend surgery as the first-line treatment for the majority of patients with appendicitis, with non-operative management reserved for specific situations in the context of shared-decision making [5, 6, 34].

This study has several limitations. As with any survey-based study, it is subject to response bias, with those holding strong opinions possibly more likely to respond. The study was limited to Denmark, and while it provides valuable insight into national practice, the findings may not be generalizable to other healthcare systems. Responses were received from the majority of hospitals and regions in Denmark. However, previous studies suggest that approximately 800 general surgeons are registered in Denmark, implying that the overall response rate for this survey was just over 25% [42]. Additionally, while the survey was rigorously developed and tested, responses were self-reported and may not perfectly reflect actual clinical behaviour. Finally, statistical corrections were not applied to account for multiple comparisons performed in the analyses of these data. As such, the statistically significant results should be interpreted with caution. Nevertheless, many of these differences are considerable and likely reflect genuine variations in clinical practices and as such this study’s findings have important implications. They suggest that while evidence and guidelines exist, their integration into daily clinical practice is inconsistent. The variation observed in our study, particularly between different regions and career stages, highlights the need for targeted interventions to harmonize care. Improved access to imaging, increased adoption of scoring systems, and the development of national consensus guidelines could help reduce unwarranted variation and promote higher-quality, more patient-centred care. At the same time, it remains essential that surgeons retain the ability to make individualised clinical decisions. Imaging, including CT, is neither fully sensitive nor specific, and timely surgical intervention is crucial for septic or clinically deteriorating patients. Balancing evidence-based protocols with sound clinical judgement is therefore key to achieving optimal outcomes in patients with suspected appendicitis. Furthermore, the misalignment between surgeons’ clinical decisions and their personal treatment preferences should prompt reflection and dialogue within the surgical community.

In conclusion, this study demonstrates considerable variation in how Danish surgeons manage patients with suspected appendicitis, with substantial discrepancies between clinical practice and personal preference. These findings underscore the need for more standardized, evidence-based approaches to the diagnosis and treatment of appendicitis, supported by equitable access to imaging, wider adoption of risk stratification tools, and better alignment between guidelines and practice. Given the lack of current national guidelines for the management of appendicitis in Denmark, these provide an argument for increased efforts to standardise care either through guideline development or educational programmes to align practice with current evidence.

## Supplementary Information

Below is the link to the electronic supplementary material.

Supplementary Material 1

## Data Availability

The data used in this study are available upon reasonable request and following approval of the study group. Request should be made to the corresponding author. These data will be available up to 12 months after publication but extensions will be considered.
