# Supplementary material for: A National survey of surgeons’ perspectives on the treatment of adults with acute appendicitis
Source: Langenbecks Arch Surg. 2026 Jan 8;411(1):59. doi: 10.1007/s00423-025-03960-w (PMC12827304; doi:10.1007/s00423-025-03960-w)
Supplement: Supplementary file 1 — Supplementary Material 1 [file 423_2025_3960_MOESM1_ESM.docx]

**Supplementary table 1.** The survey questions distributed to surgeons routinely treating adults with suspected appendicitis.

| **Section 1 - Demographics** | |
| --- | --- |
| Is your hospital routinely referred patients with suspected appendicitis? | Yes – children and adults |
|  | Yes – adults only |
|  | No |
| Which region do you work in? | Region Hovedstaden |
|  | Region Midt |
|  | Region Nord |
|  | Region Sjælland |
|  | Region Syd |
| What grade are you? | HU1 |
|  | HU2 |
|  | HU3 |
|  | HU4 |
|  | HU5 |
|  | Afdelingslæge |
|  | Overlæge |
| What age are you? | < 30 years |
|  | 30 – 40 years |
|  | 41 – 50 years |
|  | > 50 years |

| **Section 2 - Diagnostic work-up** | |
| --- | --- |
| During daytime hours on weekdays, what best describes the availability of ultrasound for the assessment of patients with suspected appendicitis in your unit? | Routinely available |
|  | Available on request |
|  | Poorly available – other modalities preferred |
|  | Not available |
| During daytime hours on weekdays, what best describes the availability of CT for the assessment of patients with suspected appendicitis in your unit? | Routinely available |
|  | Available on request |
|  | Poorly available – other modalities preferred |
|  | Not available |
| At weekends, is ultrasound routinely available for the assessment of patients with suspected appendicitis in your unit? | Yes |
|  | No |
| At weekends, is CT routinely available for the assessment of patients with suspected appendicitis in your unit? | Routinely available |
|  | Available for urgent requests |
|  | No |
| At night, is CT routinely available for the assessment of patients with suspected appendicitis in your unit? | Routinely available |
|  | Available for urgent requests |
|  | No |
| Do you use appendicitis scoring systems in the assessment of patients with suspected appendicitis? | Always |
|  | Often |
|  | Sometimes |
|  | Rarely |
|  | Never |
| In your opinion, which of the following patients should have a CT scan prior to surgery?  (tick all that apply) | Male aged > 50 years with typical presentation |
|  | Male aged < 50 years with typical presentation |
|  | Female aged > 50 years with typical presentation |
|  | Female aged < 50 years with typical presentation |
|  | Male aged > 50 years with unclear diagnosis |
|  | Male aged < 50 years with unclear diagnosis |
|  | Female aged > 50 years with unclear diagnosis |
|  | Female aged < 50 years with unclear diagnosis |
|  | Patients with delayed presentations (>3 days) |
|  | Patients with markedly raised infection markers (CRP >100) |
|  | None of the above |
| How often do you discuss the possibility of non-operative management with patients with appendicitis? | Always |
|  | Often |
|  | Sometimes |
|  | Rarely |
|  | Never |
| In your opinion, if a macroscopically normal appendix is found during operation should it be removed? | Yes |
|  | Only if no other cause of abdominal pain is found |
|  | Only after pre-operative agreement with the patients |
|  | No |

| **Section 3 - Personal preferences** | |
| --- | --- |
| You would want to be assessed using an appendicitis scoring system if you yourself were admitted with suspected appendicitis. | Strongly agree |
|  | Agree |
|  | Neither agree or disagree |
|  | Disagree |
|  | Strongly disagree |
| You would want to undergo medical imaging to confirm the diagnosis prior to surgery if you yourself were admitted with suspected appendicitis. | Strongly agree |
|  | Agree |
|  | Neither agree or disagree |
|  | Disagree |
|  | Strongly disagree |
| You would prefer to undergo CT scanning rather than an abdominal ultrasound if medical imaging was to be performed. | Strongly agree |
|  | Agree |
|  | Neither agree or disagree |
|  | Disagree |
|  | Strongly disagree |
| You would consider non-operative management if you were diagnosed with acute uncomplicated appendicitis | Strongly agree |
|  | Agree |
|  | Neither agree or disagree |
|  | Disagree |
|  | Strongly disagree |
